# Housing, Community, and Equity: Strengthening Disaster Resilience Among Older Adults

**DOI:** 10.1093/geroni/igaf122.1428

**Published:** 2025-12-31

**Authors:** Zhirui Chen, Tam Perry

## Abstract

Climate change is intensifying disasters and disproportionately affects older adults, particularly those who are low-income, racial minorities, or living with disabilities. This symposium employs quantitative, qualitative, and mixed methods to explore disaster preparedness and response of older adults, focusing on the role of housing and neighborhood resources in shaping vulnerability and resilience. The first presentation examines the associations between housing characteristics (housing type, ownership, crowding, and cost, assessed at both the individual and community level) and disaster preparedness, highlighting the needs of older adults with low income facing housing vulnerability. The second presentation explores older adults’ and community partners’ experience with extreme heat and identifies best practices and barriers to effective response in affordable rental housing. The third presentation investigates preparedness in low-income housing, emphasizing the heightened risks for isolated or disabled older adults and presenting a toolkit to enhance disaster preparedness in affordable housing communities. The fourth presentation examines housing insecurity following climate disasters, underscoring systemic disparities affecting low-income Black and Hispanic communities. Finally, the fifth presentation examines the relationship between disasters and loneliness in older adults, as well as the mediating roles of ageism and social participation (e.g., social ties and activities of social nature). Together, these studies highlight the critical need for targeted, neighborhood-level interventions to enhance disaster preparedness and resilience among older adults. The symposium will conclude with implications for research, practice, and policy, advocating for equitable resource distribution, improved housing policies, and community-based strategies to support aging populations in disaster contexts. Disasters and Older Adults Interest Group Sponsored Symposium

